# Central nervous system symptoms in mpox patients without HIV co-infection: a meta-analysis

**DOI:** 10.3389/fmed.2025.1672518

**Published:** 2025-12-09

**Authors:** Junwen Luan, Weimiao Lv, Xiaoyan Wang, Yuxuan Du, Xuejia Wang, Leiliang Zhang

**Affiliations:** 1 Department of Clinical Laboratory Medicine, The First Affiliated Hospital of Shandong First Medical University & Shandong Provincial Qianfoshan Hospital, Jinan, Shandong, China; 2 Department of Pathogen Biology, School of Clinical and Basic Medical Sciences, Shandong First Medical University & Shandong Academy of Medical Sciences, Jinan, Shandong, China

**Keywords:** mpox, MPXV, central nervous system symptoms, meta-analysis, neurological manifestations

## Abstract

**Background:**

Co-infection with HIV in patients infected with the mpox virus (MPXV) may pose a potential risk of influencing neurological manifestations. We aimed to synthesize the central nervous system (CNS) symptoms reported by mpox patients without HIV co-infection.

**Methods:**

We conducted a search for studies on mpox published in PubMed, Embase, Web of Science, Cochrane, CNKI, Wanfang Data, and Weipu Data till 2023/11/19. The type of studies included were cohort studies, case series, case–control trials, and randomized controlled trials. Reviews, editorials, pre-prints, and conference proceedings were excluded. We only included studies that reported neurological manifestations in mpox patients without HIV. STATA (version 16.0) was used to create forest plots that pooled the mean scores of CNS symptoms in mpox patients. Outcomes were presented with 95% confidence interval (95% CI), and heterogeneity was assessed using the *I*^2^ statistic.

**Results:**

We identified 2,311 unique studies, of which 14 (with a total of 989 patients) met the inclusion criteria. The quality of each study was assessed using the Newcastle-Ottawa Scale (NOS) and the Agency for Healthcare Research and Quality (AHRQ) tool. The majority of the included articles were rated as medium quality. The major CNS clinical features were myalgia, headache, and fatigue/asthenia, with pooled mean scores of 0.22 (95% CI: 0.13–0.31), 0.25 (95% CI: 0.17–0.33) and 0.28 (95% CI: 0.21–0.37) in mpox patients without HIV. The results of the sensitivity analysis showed that the meta-analysis results were stable. Additionally, Egger’s test (*p* < 0·05) suggested that a low risk of publication bias in the included literature.

**Conclusion:**

The MPXV may cause clinical nervous system injuries, including headache, myalgia, and fatigue/asthenia, as observed in approximately 25% (±3%) of the studied population.

**Systematic review registration:**

https://www.crd.york.ac.uk/PROSPERO/view/CRD42024464199, CRD42024464199.

## 1 Introduction

On 23 July 2022 and again on 14 August 2024, the World Health Organization (WHO) declared the ongoing mpox outbreak a public health emergency of international concern (PHEIC). The mpox virus (MPXV), which belongs to the *Orthopoxvirus* genus of the *Poxviridae* family, is a double-stranded DNA virus that affects humans and livestock. In 1958, MPXV was first discovered in monkeys (1). The first detected human case of mpox infection occurred in a 9-month-old child who had not received a smallpox vaccination in the Congo in 1970 (2). Following this initial case, mpox was mainly limited to epidemics and outbreaks in some countries in Central and Western Africa (3, 4). Furthermore, the recent mpox outbreak mainly affected bisexual men who have sex with men (MSM) or explicitly identified gay individuals (5). The majority of mpox patients predominantly presented with systemic symptoms that included fever, skin rash, headache, lymphadenopathy, and myalgia, among others (6).

Interestingly, patients with MPXV have recently been found to exhibit a number of neurological symptoms, including headache, myalgia, fatigue, nausea, vomiting, and other manifestations. In fact, it has been previously reported that MPXV can infect nerve cells through the olfactory epithelium and can spread through infected monocytes/macrophages in animals (7). In addition, animal studies have revealed that MPXV is capable of crossing the blood–brain barrier (BBB) and reaching brain tissue (8). However, it is reported that 38–50% of mpox patients have HIV infection (9). Furthermore, previous studies have suggested that early HIV infection can also cause central nervous system (CNS) manifestations in early stages (10). This raises the question of whether the clinically observed CNS symptoms are caused by MPXV or HIV.

To date, CNS symptoms in mpox patients, whether HIV-positive or HIV-negative, remain poorly understood and characterized due to an evident lack of literature. Accordingly, in this meta-analysis, we focus exclusively on mpox cases that are HIV-negative to eliminate the effect of HIV co-infection and comprehensively confirm whether the clinical neurological manifestations are caused by MPXV.

## 2 Methods

### 2.1 Search strategy and selection criteria

The study protocol for this meta-analysis followed the PRISMA guidelines and was registered in PROSPERO (CRD42024464199). Electronic databases that included PubMed, Embase, Web of Science, Cochrane, CNKI, Wanfang Data, and Weipu Data were searched for articles published till 2023/11/19. Keywords included (“mpox” OR “monkeypox” OR “monkeypox virus”) AND (“fever” OR “chill” OR “myalgia” OR “headache” OR “fatigue” OR “asthenia” OR “malaise” OR “pruritus” OR “nausea” OR “vomit” OR “photophobia” OR “altered conscious” OR “agitation” OR “anorexia”). Details of the search strategy are presented in Supplementary material. We included studies that reported the prevalence of at least two CNS clinical features in HIV-negative mpox patients, provided that there were at least 10 cases. There were no language-based exclusion criteria, and participants could be of any age and ethnicity as long as they were infected with mpox. Cohort studies, case series, case–control trials, and randomized controlled trials were included. The exclusion criteria are as follows: (1) duplicate publications; (2) studies that are reviews, books, documents, and meta-analysis papers; (3) articles without accessible full text; (4) single case reports and studies involving fewer than 10 MPXV-infected individuals; and (5) articles that are not related to mpox or those focusing on HIV-negative individuals. We only used data included in the articles, and we did not contact the authors of the included research. We also did not search trial registry platforms or request information from any unpublished studies identified. Screening of titles and abstracts for each article was conducted independently by two authors. Subsequently, potential qualifying studies were identified by examining full texts. Any disagreement between the two authors was arbitrated by a third author.

### 2.2 Data analysis

Two authors independently screened citations using a standardized form with predetermined inclusion and exclusion criteria and extracted data. Duplicate studies were excluded, and two independent reviewers screened the titles and abstracts of the identified research articles. They then reviewed the full text of the studies that met the eligibility criteria. We excluded studies with fewer than 10 cases to reduce publication bias. For studies that met the inclusion criteria, we extracted the following data: (1) first author; (2) publication year; (3) start and end year of inclusion; (4) study design; (5) sample size; (6) characteristics of cases (sex assigned at birth, age, country, ethnicity, sexual orientation including heterosexual, MSM, and bisexual, and HIV status); and (7) the outcome of interest: the proportion of any CNS clinical features in humans infected with MPXV.

Two reviewers employed quality assessment tools to evaluate the included studies. The quality of the included cohort studies was evaluated using the Newcastle-Ottawa Scale (NOS) (11), while the Agency for Healthcare Research and Quality (AHRQ) tool was used for the appraisal of cross-sectional studies (12). Utilizing NOS scores, the included studies were categorized as follows: overall scores of 0–3 indicate poor quality, 4–6 indicate fair quality, and 7–9 indicate good quality. The AHRQ tool has 11 items; a score of 1 is given for each item that is determined to be “Yes,” and a score of 0 is given for “No” or “Unclear.” Studies with total scores of 0 to 3 are considered low quality. Those with scores between 4 and 7 are typically regarded as medium quality, while scores ranging from 8 to 11 are classified as high quality. If two reviewers could not reach an agreement, a third reviewer would independently evaluate the study to resolve the conflict.

Each included study was summarized under the following headings: study design, participants (sample size, age, sex assigned at birth, ethnicity), and CNS manifestations. The data were then organized into a table. We collected the clinical data of mpox patients, of which the HIV-negative patients were extracted for further analysis. The medians and interquartile ranges of continuous data were converted to means and standard deviations. Forest plots were used to display the proportion of CNS symptoms reported in each study, along with the overall pooled prevalence estimates. Additionally, all analyses were performed using STATA (version 16·0), and the outcomes were presented with 95% confidence interval (95% CI). For all meta-analyses, the Cochrane Q, *p*-value, and *I*^2^ statistic were applied to check heterogeneity. When the *p*-value is greater than 0.1 or I^2^ is less than 50%, indicating no significant heterogeneity among the studies, the fixed-effect model was used to combine the data. Otherwise, a random-effects model was used (13), followed by further subgroup analysis. The reliability and robustness of the model were evaluated through sensitivity analysis. Additionally, we investigated the presence of publication bias using Egger’s test (14), with a *p*-value of less than 0.05 indicating the presence of publication bias.

## 3 Results

**Figure 1:**
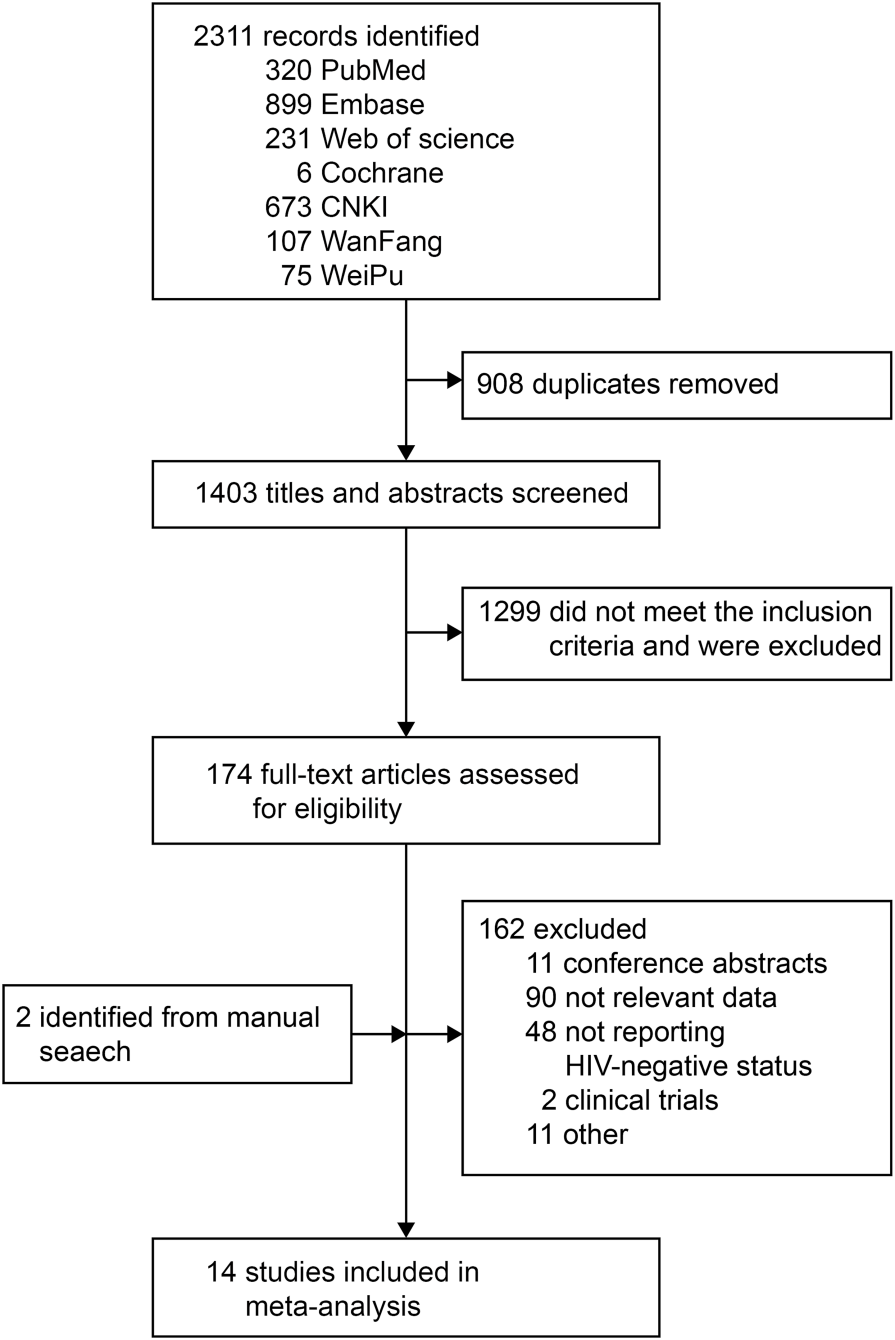
PRISMA flow chart illustrating the selection procedure of the included studies.

Seven databases, namely PubMed, Embase, Web of Science, the Cochrane Library, CNKI, Wanfang Data, and Weipu Data, were initially retrieved to obtain 2,311 potentially relevant articles. After the automatic and manual removal of duplicates, 2 independent authors screened the titles and abstracts of 1,403 studies and assessed the eligibility of the full text for 178 potential articles. Finally, based on the inclusion and exclusion criteria, 14 articles were eligible for inclusion in the meta-analysis (15–28). The full literature retrieval and screening process are presented in Figure 1. Overall, the articles included were published between 2022 and 2023, confirming a total of 989 cases of individuals with MPXV who did not have HIV infection. The sample size ranged from 10 to 266 participants. The characteristics of the included studies are summarized in Table 1. All studies confirmed mpox infection through nucleic acid tests (NATs), except for one article that did not mention it (15). Furthermore, the studies focused on adult patients. The median age across nine studies varied from 25.5 to 37 years (*n* = 447), with the majority of the participants being men. The study populations primarily consisted of patients drawn from hospitals or centers for infectious diseases (17–21, 23, 24, 27, 28), laboratories (15, 25, 26), health systems (22), and the GeoSentinel Network (16). Then, the sample size ranged from 10 to 266 patients with mpox infection who did not have HIV. In terms of geographical distribution, the majority of the studies were conducted in Europe, Asia, North America, and South America.

**Table 1:** Baseline study characteristics of mpox patients without HIV included in the study.

Study	Location	Study population	Study type	Specimen detection	*N*	Time period of data collection	Age, years mean ± SD/median (IQR)	Sex (male/female)	Ethnic group	Neurological manifestations
Zaqout et al. (28)	Qatar	Communicable Diseases Center in Doha, Qatar	Observational study	PCR	12	13 May 2022–30 November 2022	33·5(24·5–37·5)	10/2	··	Fever 7/12, myalgia 3/12, fatigue/asthenia 3/12
Kowalski et al. (23)	Central Europe	Hospital for Infectious Diseases in Warsaw, Poland	Cohort study	RT-PCR	48	16 May 2022–30 October 2022	32 (26–37)	48/0	··	Fever 37/48, headache 4/48, myalgia 13/48, fatigue/asthenia/malaise 26/48
Silva et al. (27)	Brazil	A major referral center for Infectious Diseases in Rio de Janeiro, Brazil	Cohort study	RT-PCR	96	12 June 2022–19 August 2022	31 (25–37·5)	91(Cisgender men)/5(Cisgender women)	Black 34/65, Pardo (mixed) 14/65, White 18/65	Fever 50/96, headache 32/96, myalgia 16/96, fatigue/asthenia 23/96, nausea 2/96
Kim et al. (22)	USA	Two large health systems in South Florida	Retrospective observational study	PCR	98	01 January 2020–10 September 2022	33 (28–42)	89/9	White 68/98, Black 24/98, Other 6/98	Fever 33/98, headache 3/98, myalgia 18/96, fatigue/asthenia/malaise 13/96
Pilkington et al. (26)	UK	Department of Sexual Health at King’s College Hospital in South-East London, UK	Retrospective observational study	PCR	86	May 2022–December 2022	34 (29–40)	85/1	White 11/86, Non-White 14/86, Unknown 61/86	Fever 52/86, headache 18/86, myalgia 14/86
Jun et al. (21)	China	Hangzhou Xixi Hospital	Retrospective study	NAT	10	15 Jun 2023–05 August 2023	25·5 (23–31)	10/0	Yellow 10/10	Fever 7/10, headache 3/10, myalgia 2/10, fatigue/asthenia 1/10
Chen et al. (18)	China	Shenzhen Third People’s Hospital	Retrospective study	PCR	19	09 June 2023–08 July 2023	··	19/0	Yellow 19/19	Fever 16/19, headache 3/19, myalgia 6/19, fatigue/asthenia 3/19
Núñez et al. (25)	Mexico	National epidemiologic case report	Observational study	RT-RCR	266	24 May 2022–05 September 2022	··	250/16	··	Fever 205/266, headache 112/266, myalgia 119/266, fatigue/asthenia 85/266, nausea 5/266, vomiting 4/266
Hoffmann et al. (20)	Germany	42 German centers	Retrospective cohort study	PCR	58	19 May 2022–30 June 2022	37	58/0	··	Fever 28/52, headache 19/52
Caria et al. (17)	Portugal	A Portuguese hospital	Retrospective observational study	NAAT	16	05 May 2022–26 July 2022	31·0 (8·0)	15/1	··	Fever 8/16, headache 1/16, myalgia 2/16, fatigue/asthenia 2/16
Fu et al. (19)	China	six major infectious disease hospitals and one Center for Disease Control and Prevention in China	Cross-sectional study	PCR	50	01 June 2023–31 July 2023	··	50/0	Yellow 50/50	Fever 36/50, headache 8/50, myalgia 10/50, fatigue/asthenia 10/50
Angelo et al. (16)	GeoSentinel Network (29 countries)	71 clinical sites in 29 countries	Cross-sectional study	PCR	134	01 May 2022–01 July 2022	··	134/0	··	Fever 75/134, headache 20/134, myalgia 19/134, fatigue/asthenia malaise 57/134, nausea 2/134
Kowalski et al. (23)	Portugal	··	Retrospective study	··	20	··	32·5 ± 8·1	20 (cisgender men)/0	··	Fever 12/20, headache 10/20, myalgia 12/20
Maronese et al. (24)	Italy	Five dermatology referral centers in Italy	Retrospective study	PCR	76	01 June 2022–31 October 2022	··	··	··	Fever 47/76, headache 17/76, myalgia 23/76, fatigue/asthenia 29/76

PCR, polymerase chain reaction; RT-PCR, real-time PCR; NAT, nucleic acid test; NAAT, nucleic acid amplification test.

A total of 12 observational cohort studies were assessed using the NOS quality assessment tool, while 2 cross-sectional studies were evaluated using the AHRQ tool. Our results are summarized in Supplementary Tables S1, S2. The analysis of the existing literature revealed that only one study was of high quality (27), 12 studies were of medium quality (16–26, 28), and one study was of low quality (15). The majority of cohort studies lost points on comparability in the NOS due to the absence of a control group. In the AHRQ tool, the risk of bias stemmed mainly from items 2, 5, 7, 8, 9, and 11.

**Figure 2:**
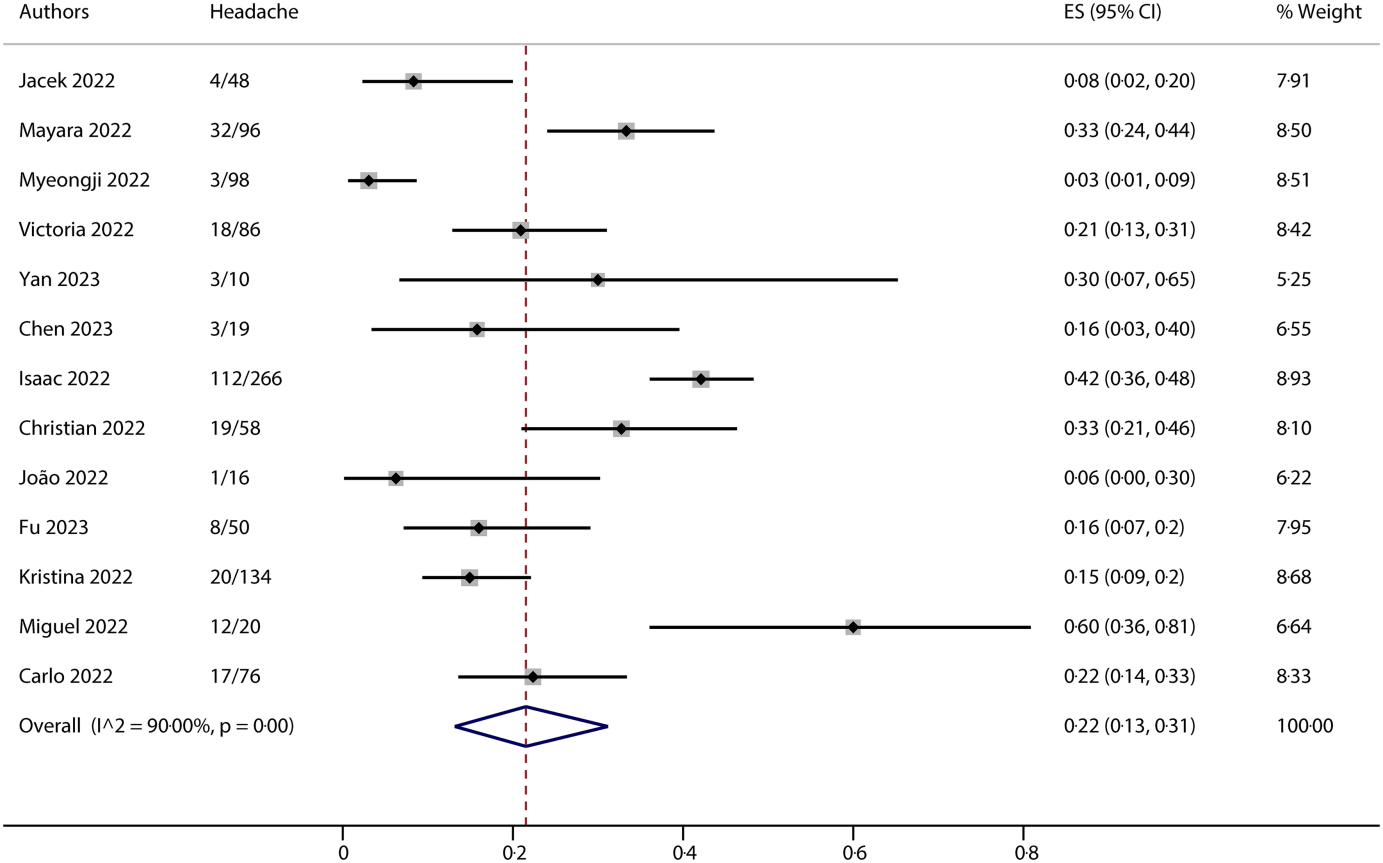
Forest plot of headache and comparative character in mpox patients without HIV co-infection.

**Figure 3:**
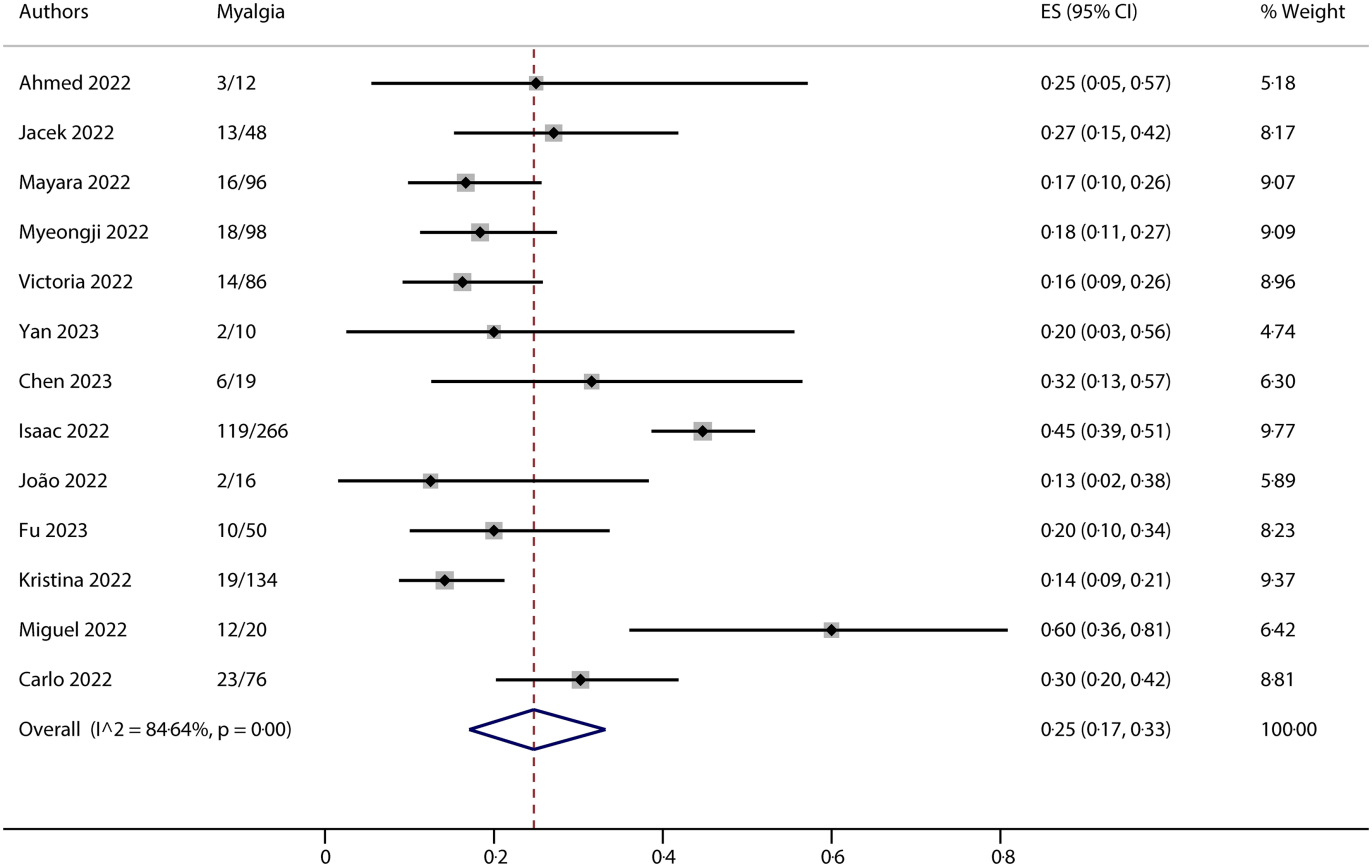
Forest plot of myalgia and comparative character in mpox patients without HIV co-infection.

**Figure 4:**
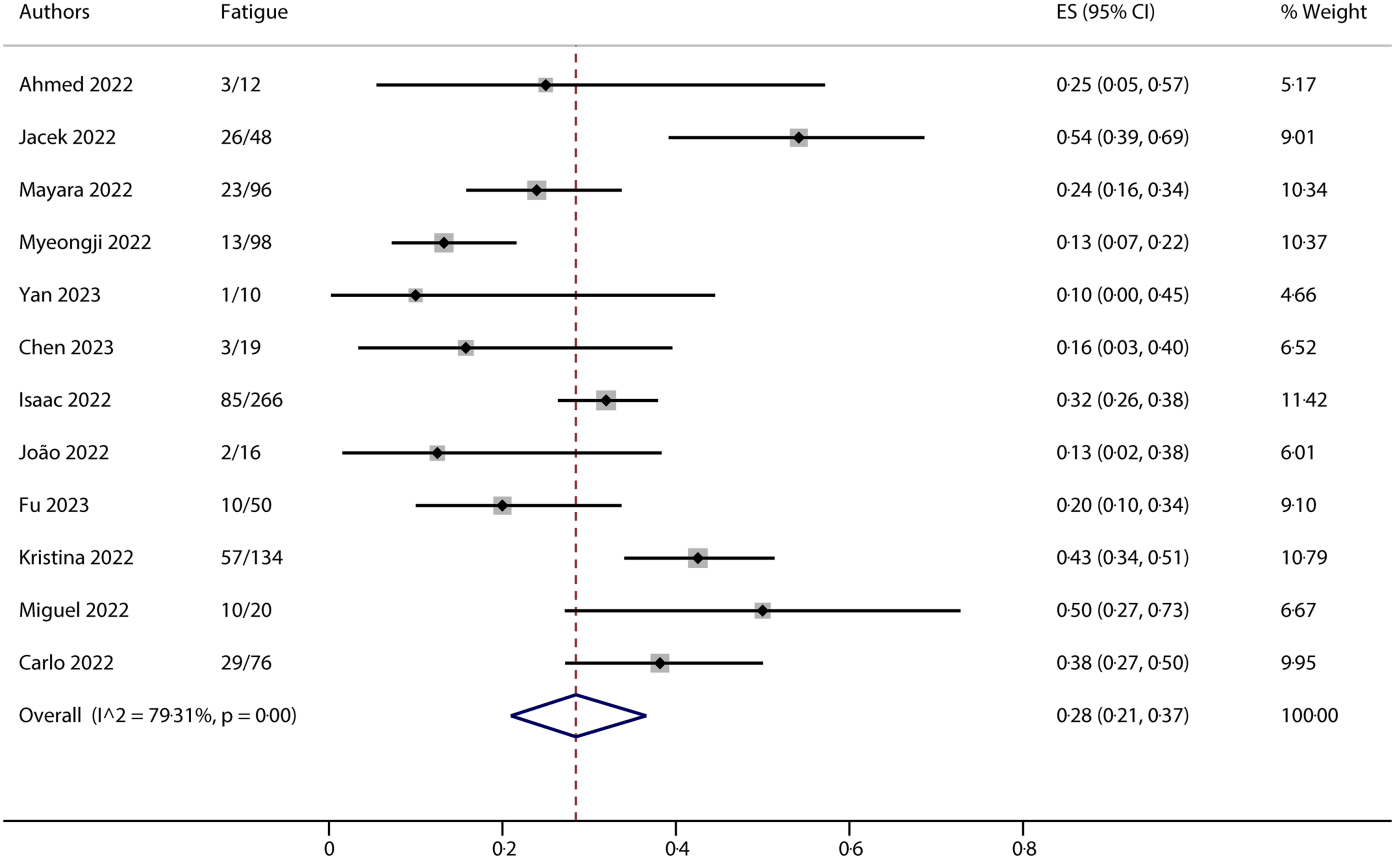
Forest plot of fatigue/asthenia and comparative character in mpox patients without HIV co-infection.

**Figure 5:**
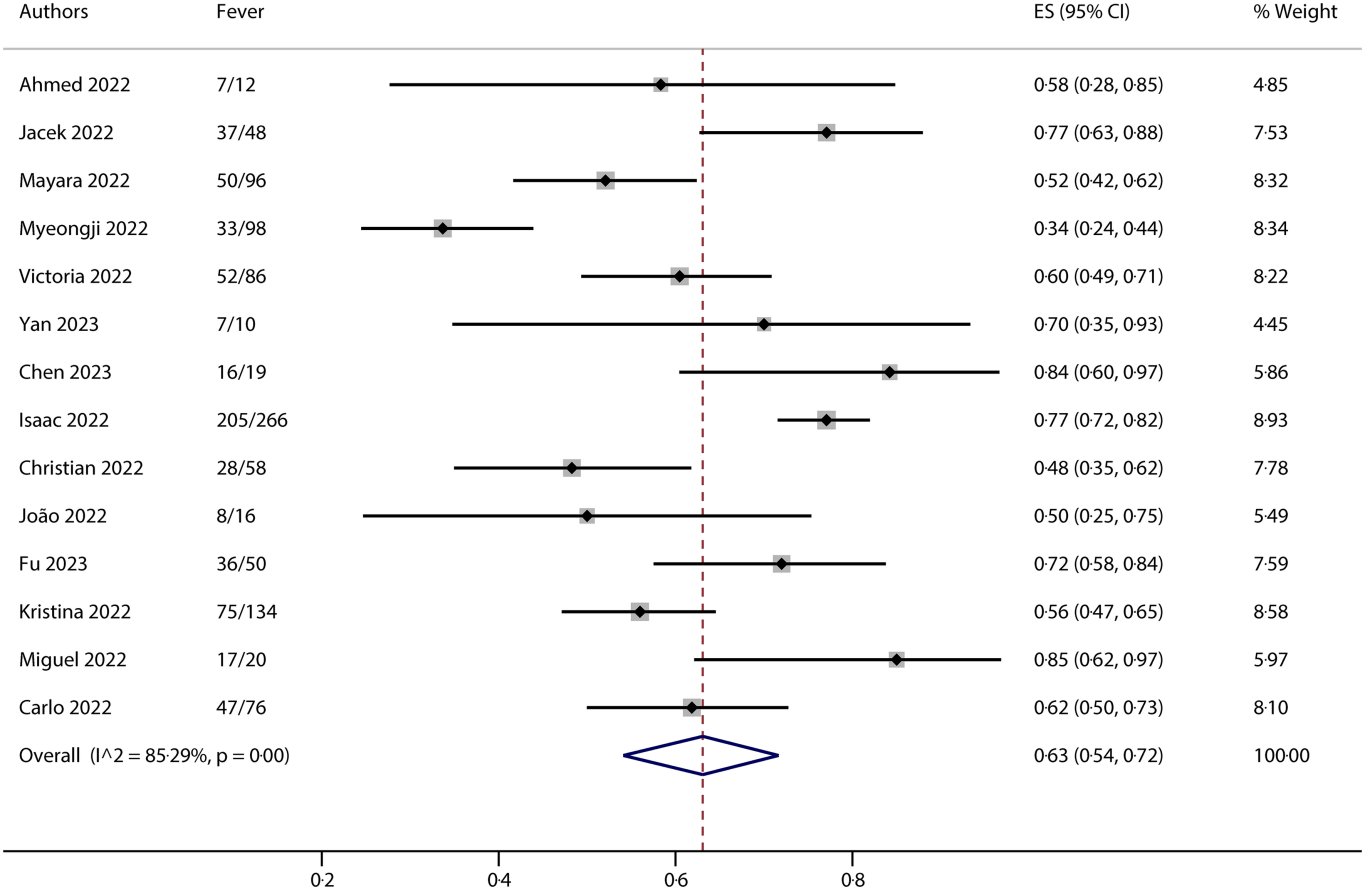
Forest plot of fever and comparative character in mpox patients without HIV co-infection.

All studies included in this meta-analysis contained at least one neurological manifestation. A total of 14 studies were included in the analysis. The neurological manifestations were headache (13 studies), myalgia (13 studies), and fatigue/asthenia (12 studies), whereas fever (14 studies) was a comparative characteristic of non-neurological manifestations for mpox infection. The metaprop (version 1.06) was used to pool the effects and conduct the analysis. Forest plots demonstrated that all meta results of these characteristics exhibited substantial heterogeneity, with I^2^ ranging from 79.31 to 90.00% (Figures 2–5). Therefore, the random-effects model was employed to pool the mean scores, which were presented with weighted effect sizes and 95% CI. The mean pooled scores for headache, myalgia, fatigue/asthenia, and fever were 0.22 (95% CI: 0.13–0.31) (Figure 2), 0.25 (95% CI: 0.17–0.33) (Figure 3), 0.28 (95% CI: 0.21–0.37) (Figure 4), and 0.63 (95% CI: 0.54–0.72) (Figure 5) in mpox patients without HIV, respectively.

The results of this meta-analysis suggested that there was high heterogeneity in clinical characteristics. For exploring potential sources of heterogeneity, we conducted a series of subgroup analyses for each of the clinical characteristics. The entire group is divided into different subgroups based on different factors, such as region, ethnicity, gender, and MSM ratio. The subgroup analysis was also conducted using metaprop (version 1.06), which indicated that the region might be the most important factor contributing to the observed heterogeneity (any subgroup containing no more than three studies would yield no results in the metaprop calculations). For headache, the total *I*^2^ is 90.00%, while it is 76.02% in the Europe subgroup. Although there are no results for the Asia, America, and Mixed subgroups because each contains three or fewer studies, we observed relatively good consistency across the different subgroups (Supplementary Figure S1A). For myalgia, the total *I*^2^ is 84.64%, while it is 0% in the Asia subgroup. As shown in Supplementary Figure S1B, there is a notable absence of outcomes in other subgroups. For fatigue/asthenia, the total *I*^2^ is 79.31%, but again, it is 0% in the Asia subgroup, with relatively good consistency observed in the other subgroups without results (Supplementary Figure S1C). Regarding the presence of fever, the total *I*^2^ is 85.29%. However, in the Asia and Europe subgroups, the values are 0% and 67.02%, respectively. The data indicated relatively good consistency in the American and Mixed subgroups, which also did not yield results (Supplementary Figure S1D).

We performed Egger’s test to assess the publication bias, and the results are presented in Supplementary Figure S2. The analysis of clinical research indicators in the literature showed that there was no risk of publication bias for each characteristic. The *p*-values for headache, myalgia, fatigue/asthenia, and fever were 0.089, 0.827, 0.930, and 0.543 (*p* < 0.05), respectively. The results of the bias analysis implied that there were no small-study effects in the analysis.

A sensitivity analysis was performed to evaluate the reliability and robustness. The results of the sensitivity analysis indicated that heterogeneity was not significantly impacted by the literature data selected for the meta-analysis. The conclusion was found to be relatively stable (Supplementary Figure S3).

## 4 Discussion

The most frequently reported neurological manifestations of human mpox were headache, myalgia, fatigue, and photophobia. Furthermore, MPXV can lead to potentially fatal illnesses, including seizures, encephalitis, visual deficits, and coma (29). It was found that MSM, particularly those infected with HIV, were the most affected group when having mpox infections (30). Any possible correlation between MPXV and CNS manifestations may potentially be further complicated by the impact of co-infection with HIV. Our meta-analysis aimed to explore the CNS symptoms of mpox patients who were not infected with HIV in order to exclude the effects of HIV on neurological manifestations. Studies including more than 10 cases of mpox without HIV were selected for this research. Overall, the results of the meta-analysis regarding neurological manifestations indicated that MPXV can indeed lead to clinical signs of nervous system infection. The findings of our pooled analysis revealed that the most prevalent CNS symptoms reported among patients infected with monkeypox were headache, myalgia, and fatigue/asthenia. In addition, the findings of the meta-analysis demonstrated strong consistency in the penetrance of three CNS symptoms, which ranged from 22 to 28%, suggesting that the overall rate of patients with clinical neurological symptoms of MPXV infection was approximately 25% (±3%). Notably, as an autoimmune clinical manifestation rather than a CNS symptom, fever symptom result manifests that the consistency of nervous system traits is not a random and accidental phenomenon. This highlights the significant impact of MPXV on the nervous system, indicating a need for further clinical attention. Additionally, the neurological manifestations observed are heterogeneous, with *I*^2^ values ranging from 79.31% to 90.00%. A subgroup analysis was conducted, which reduced the heterogeneity observed. These findings suggest that a particular region may be the primary contributor to the observed variability. The differences are likely related to distinct MPXV clades across various geographical regions, differing modes of transmission, and the inclusion of studies with limited sample sizes. Other understudied factors that may contribute to this heterogeneity include significant variations within regions. Some regions studied were grouped, and the ethnic demographics included individuals from diverse ethnic backgrounds, such as Indians, Europeans, and South Americans, which may also have contributed to the observed diversity.

Notably, the exact pathophysiology of the neuroinvasive and neurotropic nature of MPXV remains to be elucidated. However, studies conducted using animal models have suggested two probable mechanisms: (i) the olfactory epithelium route and (ii) the infection of macrophages/monocytes, which may allow the virus to permeate brain tissue by crossing the BBB (7, 8). It is noteworthy that a recent article emphasizes the potential brain cell tropism and neurovirulent activity of MPXV. The study highlights that MPXV preferentially infects astrocytes, causing the proteolytic cleavage of gasdermin B (GSDMB), which is an essential stage in the process of pyroptosis. Furthermore, this infection results in the death of inflammatory cells. Interestingly, treatment with dimethyl fumarate (DMF) has been shown to reduce MPXV infection and cell death (31). These findings provide possible treatment alternatives and shed light on the previously identified neuropathogenic effects of MPXV in humans. Further research is necessary to explore the specific routes by which the MPXV invades the brain as well as to understand the neurological manifestations experienced by mpox patients. It is important to note that the clinical manifestations of mpox and smallpox show significant similarities, particularly regarding neurological effects (32). Patients present common signs such as headache and lymphadenopathy during the prodromal phase of both mpox and smallpox (33). While mpox can lead to a range of neurological manifestations and very rarely results in encephalitis, encephalitis caused by smallpox is linked to significant morbidity and mortality (32). Further research is required to elucidate the nuances of neurological complications in both infections. Recently, there are no fully approved treatments or vaccines for MXPV. Since mpox and smallpox share a genetic resemblance, antiviral medications against orthopoxviruses such as tecovirimat, brincidofovir, and cidofovir, along with vaccinia immune globulin (VIG) and supportive care, can be used to treat mpox infection. ACAM2000 and JYNNEOS vaccinations have been suggested as possible prophylactic measures for adults who are highly susceptible to contracting smallpox and mpox (34). Interestingly, one recent study summarized the potential regulatory role of microRNA in the CNS during mpox infection, indicating that microRNAs could also be a possible target to treat mpox CNS symptoms (35). Further research is recommended to explore improved strategies and identify potential drug targets.

However, this meta-analysis does have some limitations. In terms of assessing the quality of the included studies, 13 of the studies analyzed were rated as having medium or low quality, highlighting the relatively underexplored nature of CNS symptoms in the context of the MPXV outbreak. In addition, the predominance of retrospective studies and the absence of a randomized controlled trial limited the relevance of our findings. Notably, there is a risk that neurological manifestations may not be accurately documented and reported. Comparatively, only 63% of MPXV infections were associated with fever, suggesting that a larger proportion of MPXV patients may actually have a nervous system infection. Establishing criteria for neurological severity is a challenging endeavor. Another potential limitation is that few articles have reported the diagnosis of neurological symptoms before the onset of the MPXV infection. Furthermore, patients with MPXV experienced other uncommon neurological manifestations such as nausea and vomiting. Nevertheless, due to a lack of sufficient research, we were unable to assess this phenomenon. Overall, our ability to synthesize data was hindered by factors such as the clade of MPXV, the ethnicity of mpox patients without HIV, and the uncertainty regarding the severity of CNS manifestations. In summary, our analysis presents the classical CNS symptoms associated with MPXV, including myalgia, headache, and fatigue/asthenia, and emphasizes the need for clinical attention to the neurological symptoms caused by mpox.

## 5 Conclusion

This meta-analysis demonstrates that MPXV can lead to significant neurological symptoms, with approximately 25% of patients exhibiting CNS manifestations, such as headache, myalgia, and fatigue/asthenia. The findings suggest that these neurological presentations are attributed to MPXV rather than HIV. However, further prospective studies are necessary to achieve a more comprehensive understanding and quantification of the severity of these clinical neurological manifestations.

## Data Availability

The original contributions presented in the study are included in the article/Supplementary material, further inquiries can be directed to the corresponding author.
