# Supplementary material for: Central nervous system symptoms in mpox patients without HIV co-infection: a meta-analysis
Source: Front Med (Lausanne). 2025 Dec 9;12:1672518. doi: 10.3389/fmed.2025.1672518 (PMC12723029; doi:10.3389/fmed.2025.1672518)
Supplement: Supplementary file 1 [file Data_Sheet_1.pdf]

Supplementary Material

Central Nervous System Symptoms in mpox Patients without HIV Co-infection: A Meta-Analysis

Junwen Luan, Weimiao Lv, Xiaoyan Wang, Yuxuan Du, Xuejia Wang, Leiliang Zhang

Table of contents

Supplementary Material 1. Search strategy **1**
Supplementary Material 2. Quality Assessment with Newcastle-Ottawa Scale **2**
Supplementary Material 3. Quality assessment with AHRQ **4**
Supplementary Material 4. Forest plot of sub-group analyses **6**
Supplementary Material 5. Bias test for each character. **7**
Supplementary Material 6. Sensitive analyses. **8**

Search strategy

A systematic search of the literature was performed by using PubMed, Embase, Web of Science, Cochrane, CNKI, Wanfang Data, and Weipu Data up to November 19, 2023.

- Search in PubMed

((((((((((((((fever) OR (chill)) OR (myalgia)) OR (headache)) OR (fatigue)) OR (asthenia)) OR (malaise)) OR (pruritus)) OR (nausea)) OR (vomit)) OR (photophobia)) OR (altered consious)) OR (agitation)) OR (anorexia)) AND (((mpox) OR (monkeypox)) OR (monkeypox virus)) Filters: from 1000/1/1 - 2023/11/19 (320)

- Search in Embase

1. mpox:abti OR monkeypox:abti (4736)
2. fever OR chill OR myalgia OR asthenia OR headache OR fatigue OR malaise OR pruritus OR nausea OR vomit OR (altered AND conscious)OR agitation OR anorexia OR photophobia (1533233)
3. 1 AND 2 AND [01-01-1000/sd NOT[20-11-2023]/sd (899)

- Search in Web of Science

1. TS=(mpox) OR TS=(monkeypox) OR TS=(monkeypox virus) (3848)
2. (((((((((((((ALL=(fever)) OR ALL=(chill)) OR ALL=(myalgia)) OR ALL=(asthenia) OR ALL=(headache)) OR ALL=(fatigue)) OR ALL=(malaise)) OR ALL=(pruritus)) OR ALL=(nausea)) OR ALL=(vomit)) OR ALL=(altered conscious)) OR ALL=(agitation)) OR ALL=(anorexia)) OR ALL=(photophobia) (621346)
3. 1 AND 2 (237)
4. 3 AND Publication Date：From 1985-01-01 to 2023-11-19 (231)

- Search in Cochrane

1. (mpox) OR (monkeypox)) OR (monkeypox virus) (36)
2. (fever) OR (chill)) OR (myalgia)) OR (headache)) OR (fatigue)) OR (asthenia)) OR (malaise)) OR (pruritus)) OR (nausea)) OR (vomit)) OR (photophobia)) OR

(altered consious)) OR (agitation)) OR (anorexia) (156080)

3. 1 AND 2 with Cochrane Library publication date from Jan 1000 to Nov 2023 (6)

- Search in CNKI (in Chinese and English)

(FT=fever OR FT=chill OR FT=myalgia OR FT=asthenia OR FT=headache OR FT=fatigue OR FT=malaise OR FT=pruritus OR FT=nausea OR FT=vomit OR FT=altered conscious OR FT=agitation OR FT=anorexia OR FT=photophobia) AND (TKA=mpox OR TKA=monkeypox OR TKA=monkeypox virus) with up to 2023-11-19 (673)

- Search in Wanfang Data (in Chinese and English)

(THEME:(mpox) OR THEME:(monkeypox) OR THEME:(monkeypox virus)) AND (ALL:(fever) OR ALL:(chill) OR ALL:(myalgia) OR ALL:(asthenia) OR ALL:(headache) OR ALL:(fatigue) OR ALL:(malaise) OR ALL:(pruritus) OR ALL:(nausea) OR ALL:(vomit) OR ALL:(altered conscious) OR ALL:(agitation) OR ALL:(anorexia) OR ALL:(photophobia)) (107)

- Search in Weipu Date (in Chinese and English)

(M= mpox OR monkeypox) AND (U=fever OR chill OR myalgia OR asthenia OR headache OR fatigue OR malaise OR pruritus OR nausea OR vomit OR altered conscious OR agitation OR anorexia OR photophobia) (75)

Supplementary Material 2. Quality Assessment with Newcastle-Ottawa Scale

Table S1. Quality Assessment with Newcastle-Ottawa Scale

	Selection				Comparability		Outcome			NOS score
	Representativeness	Selection of non-exposed	Ascertainment of exposure	Outcome not present at start	Comparability on most important factors	Comparability on other risk factors	Assessment of outcome	Long enough follow-up	Adequacy of follow-up	
Ahmed 2022	*		*	*			*			4
Jacek 2022	*		*	*			*	*		5
Mayara 2022	*	*	*	*	*	*	*	*	*	9
Myeongji 2022	*		*	*			*			4
Victoria 2022	*		*				*	*	*	5
Yan 2023	*		*	*			*			4
Chen 2023	*		*	*			*			4
Isaac 2022	*	*	*	*			*			5
Christian 2022	*		*	*			*			4
Joao 2022	*		*	*			*		*	5
Miguel 2022	*			*						2
Carlo 2022	*		*	*			*			4

* indicates a domain was met. The full scores of each part are 4, 2, and 3 points, respectively. The aggregated categorisations used were 0−3 (poor), 4−6 (medium) and 7−9 (good).

Supplementary Material 3. Quality assessment with AHRQ

Table S2. Quality assessment with AHRQ

	Fu 2023	Kristina 2022
(1) Define the source of information (survey, record review)	Yes	Yes
(2) List inclusion and exclusion criteria for exposed and unexposed subjects (cases and controls) or refer to previous publications	No	No
(3) Indicate time period used for identifying patients	Yes	Yes
(4) Indicate whether or not subjects were consecutive if not population-based	Yes	Yes
(5) Indicate if evaluators of subjective components of study were masked to other aspects of the status of the participants	‥	‥
(6) Describe any assessments undertaken for quality assurance purposes (e.g., test/retest of primary outcome measurements)	Yes	Yes
(7) Explain any patient exclusions from analysis	‥	‥

(8) Describe how confounding was assessed and/or controlled	··	··
(9) If applicable, explain how missing data were handled in the analysis	··	··
(10) Summarize patient response rates and completeness of data collection	Yes	Yes
(11) Clarify what follow-up, if any, was expected and the percentage of patients for which incomplete data or follow-up was obtained	··	No
AHRQ score	5	5

AHRQ has 11 items, and a score of 1 is assigned when an individual item is evaluated as "Yes", otherwise a score of 0 is assigned. The total score is 0 to 3 for low quality, 4 to 7 for medium quality, and 8 to 11 for high quality.

Supplementary Material 4. Forest plot of sub-group analyses.

Fig. S1. Forest plot of sub-group analyses of neurological manifestations and comparative character in mpox patients without HIV co-infection.

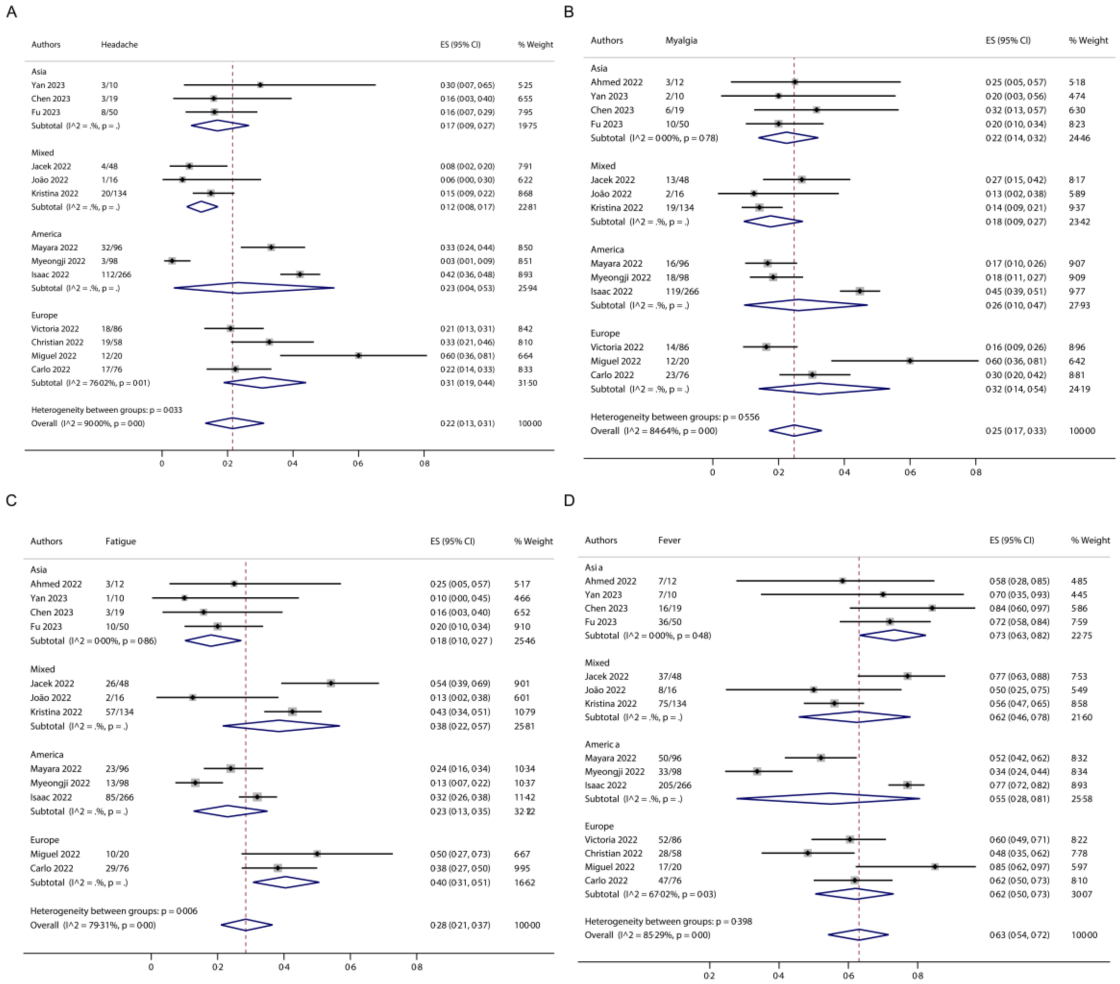

Data are presented for (A) headache, (B) myalgia, (C) fatigue/asthenia, and (D) fever.

Supplementary Material 5. Bias test for each character.

Fig. S2. The results of bias test for each character in mpox patients without HIV co-infection.

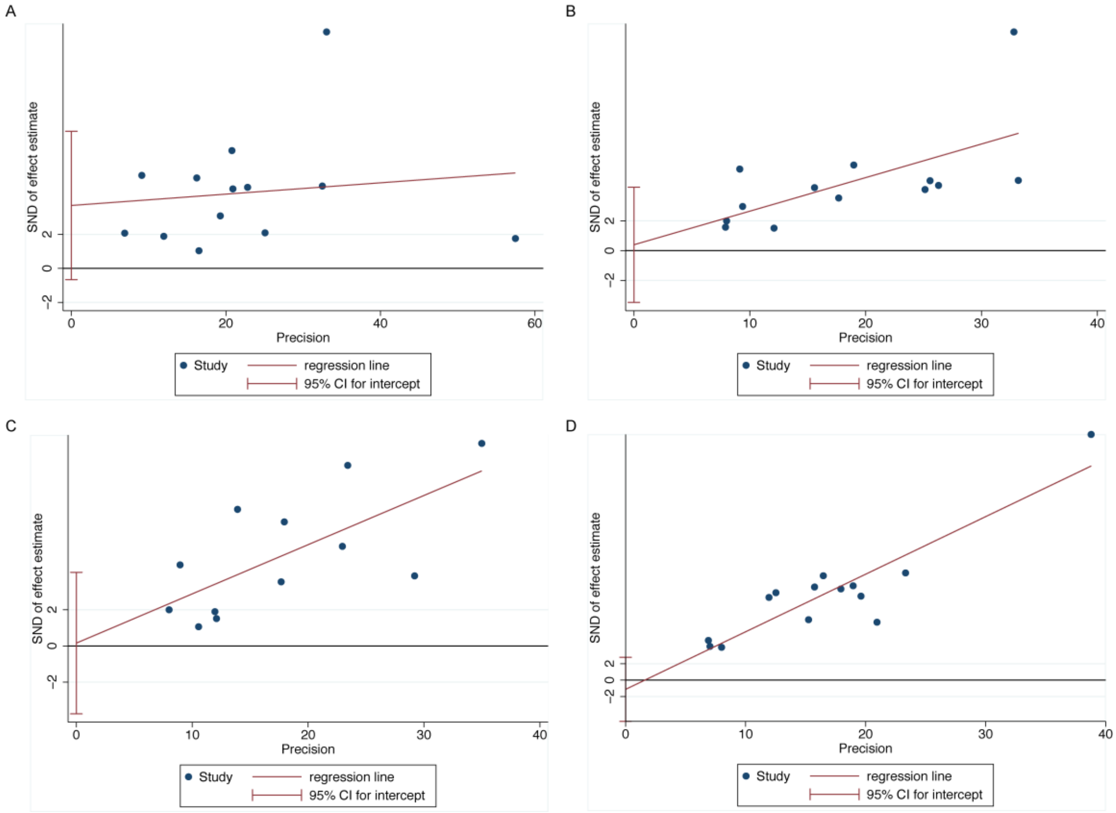

Data are presented for (A) headache, (B) myalgia, (C) fatigue/asthenia, and (D) fever.

Supplementary Material 6. Sensitive analyses.

Fig. S3. The results of sensitive analyses for each character in mpox patients without HIV co-infection.

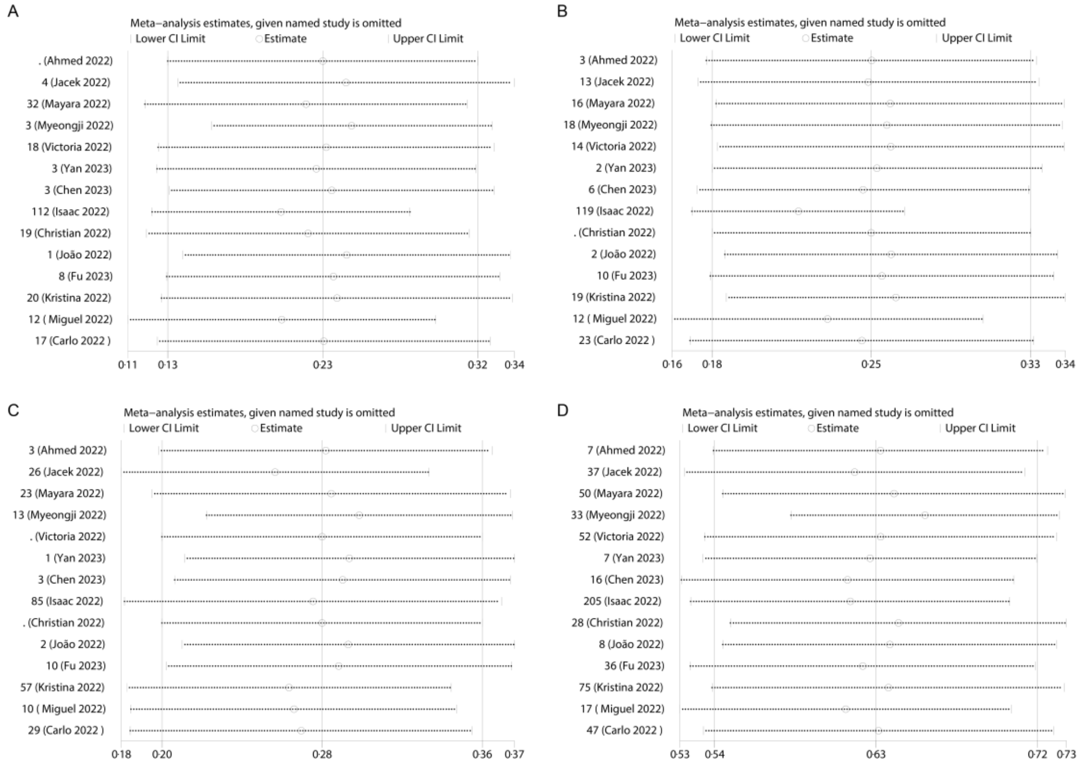

Data are presented for (A) headache, (B) myalgia, (C) fatigue/asthenia, and (D) fever.
